# Attributional explanations in preschool teachers’ competence judgments on tangram tasks: links with learning behaviors and task difficulty

**DOI:** 10.3389/fpsyg.2026.1859618

**Published:** 2026-08-05

**Authors:** Atiye Adak Özdemir

**Affiliations:** Department of Early Childhood Education, Pamukkale University, Denizli, Türkiye

**Keywords:** Preschool teachers, competence judgments, preschool children, learning behaviors, attributional reasoning

## Abstract

**Objective:**

Teachers’ competence judgments may influence children’s educational experiences, yet the factors underlying these judgments and the attributional reasoning accompanying them remain insufficiently understood. This study examined how preschool teachers evaluate children’s potential competence in tangram tasks and how these evaluations relate to children’s learning behaviors.

**Methods:**

A convergent parallel mixed-methods design was used with 195 children and 57 teachers in Türkiye.

**Results:**

As task complexity increased, the proportion of children evaluated as competent decreased significantly, and this shift was unidirectional: no child judged incapable in the simple task condition received a positive evaluation in the complex task condition. Teachers’ perceptions of children’s learning behaviors significantly predicted their competence judgments across task conditions, with attention/persistence emerging as the strongest predictor, followed by motivation, whereas learning attitude did not make a significant independent contribution. Qualitative findings, guided by Weiner’s attribution framework, showed that teachers explained their judgments predominantly through internal child-related factors. Anticipated failure among children with lower teacher-rated learning behavior scores was commonly attributed to inattention, low effort, developmental immaturity, and limited cognitive ability, whereas anticipated success among children with higher teacher-rated scores was primarily explained through effort, persistence, and ability. Contextual and instructional factors were referenced infrequently across both task conditions and learning behavior groups.

**Conclusion:**

These findings suggest that preschool teachers’ competence judgments are closely associated with teachers’ perceptions of children’s learning behaviors and are accompanied by attributional explanations that emphasize child-level characteristics over contextual factors.

## Introduction

Teachers in early childhood classrooms routinely assess children’s competencies, and these assessments have been shown to shape instructional decisions and the distribution of learning opportunities (McDermott et al., 2000). Such evaluations may reflect not only observed behavior but also teachers’ prior expectations and causal beliefs regarding children’s classroom behavior (Brophy and Good, 1970; Weiner, 1985). For instance, a teacher who observes a child persisting through a difficult puzzle or sustaining attention during a group activity may draw on these impressions when deciding how to group children, which tasks to assign, or how much support to provide. Children’s learning behaviors, including attention, persistence, and motivational engagement, have been linked to later academic achievement (McClelland et al., 2006; Razza et al., 2015) and are frequently used as cues in teachers’ assessments of academic competence. Yet whereas the association between teachers’ evaluations and children’s performance outcomes has received considerable attention (Cabell et al., 2009; Furnari et al., 2016; Südkamp et al., 2012), comparatively less attention has been directed toward the attributional reasoning underlying these evaluations, that is, how teachers explain and causally interpret what they observe, particularly in early childhood contexts. The present study addresses this gap by examining how preschool teachers evaluate children’s potential task competence, how these evaluations relate to children’s learning behaviors, and what causal reasoning teachers bring to these judgments, thereby extending prior attribution research to a prospective early childhood judgment context.

### Teacher beliefs, expectations, and attributional reasoning

Teacher beliefs, expectations, and attributions contribute to the formation of teacher judgments. Teacher beliefs, defined as subjective judgments about the truth or falsity of propositions, play a role in interpreting experiences and shaping instructional decisions (Pajares, 1992; Fives et al., 2015). Research also indicates that teacher beliefs mediate the relationship between classroom practices and children’s academic outcomes (Fang, 1996; Sabarwal et al., 2022; Spodek, 1988). Teacher expectations refer to teachers’ judgments about children’s current and future academic performance and classroom behavior (Good and Brophy, 1997). These expectations may create discrepancies between teachers’ assessments and children’s actual abilities (Jussim and Eccles, 1992; Hinnant et al., 2009; Tiedemann, 2000). Expectancy processes may operate through teacher-child interaction sequences in which teachers provide feedback and learning opportunities consistent with their beliefs (Brophy and Good, 1970; Brophy, 1983). Such processes have been associated with the emergence of achievement differences and are consistent with expectancy and self-fulfilling prophecy perspectives (Jussim and Harber, 2005; Rosenthal and Jacobson, 1968). Additionally, teacher expectations have been shown to predict later academic outcomes and influence the nature of instructional feedback (Gentrup et al., 2020).

Teachers’ instructional beliefs and practices have also been found to be related to their attributions regarding children’s behavior (Bibou-Nakou et al., 2000; Carter et al., 2014; Dobbs and Arnold, 2009; Thijs and Koomen, 2009). Attribution theory provides a framework for understanding how individuals interpret the causes of success and failure and how these interpretations influence expectations, emotions, and subsequent behavior (Kelley and Michela, 1980). According to Weiner (1985), causal explanations vary along the dimensions of locus, stability, and controllability, and these dimensions play a central role in shaping expectations of success and future behavior. In educational contexts, teachers may interpret children’s academic outcomes in attributional terms, linking success or failure to internal or external, stable or unstable causes. Attributions to stable factors may lead teachers to expect similar outcomes across tasks, whereas attributions to more controllable or unstable factors may support adaptive instructional responses and reinforce differential treatment over time.

These processes become particularly pronounced in school settings, and expectations regarding children’s behavior may take on distinct forms in early childhood settings, where behavioral readiness and self-regulation are primary concerns. In early childhood classrooms, behaviors such as following instructions, sustained attention, and adaptive engagement are often seen as indicators of success (Lane et al., 2007). Consequently, teachers may develop expectations that emphasize academic readiness, self-regulation, and task completion. In preschool classrooms, teachers may rely on observable behavioral indicators such as attention, perseverance, and engagement when forming judgments. These behaviors, often conceptualized as learning behaviors, are related to children’s academic abilities (Yen et al., 2004). Although teacher assessments can demonstrate considerable accuracy, their precision varies across domains and contexts (Hosoya et al., 2021; Urhahne and Wijnia, 2021; Li et al., 2025). In early childhood settings, teachers may both overestimate and underestimate children’s academic abilities relative to standardized assessments (Kowalski et al., 2018; McKevett and Kiss, 2019), and these assessments may not always fully reflect children’s true abilities (Gentrup et al., 2020; Kilday et al., 2012).

Despite this research, most studies have focused on the relationship between teacher assessments and test scores, with limited attention paid to the attributional processes underlying teachers’ judgments about children’s likelihood of success in cognitively demanding tasks. To date, research has concentrated on the correspondence between teachers’ evaluations and children’s standardized test performance (Südkamp et al., 2012; Urhahne and Wijnia, 2021); however, the attributional processes through which teachers arrive at these evaluations, particularly in prospective judgment contexts where performance has not yet been observed, have received comparatively little examination, especially in early childhood settings.

### Learning behaviors and approaches to learning

In the present study, the term learning behaviors is used consistently to refer to the cluster of observable, goal-directed engagement characteristics operationalized by the Preschool Learning Behaviors Scale (McDermott et al., 2000, 2002), and is distinguished from cognitive abilities and socio-emotional outcomes. Although several overlapping terms appear in the literature, including approaches to learning, learning-related skills, and behavioral engagement, these labels converge on a common referent encompassing attention, persistence, motivation, and task orientation. Where related terms appear in cited work, they are treated as referring to substantially overlapping constructs.

Children’s learning-related engagement has been identified as a robust predictor of successful school transition (Bassok and Latham, 2017; Halliday et al., 2018). The concept of approaches to learning was introduced by Kagan et al. (1995) as a core component of school readiness and is operationalized through effortful, goal-directed actions such as motivation, sustained attention, persistence, planning, openness to novelty and risk, and cooperation, distinct from cognitive skills and socio-emotional outcomes (Fantuzzo et al., 2007; McDermott et al., 2011). These behaviors reflect cognitive, affective, and behavioral characteristics associated with self-regulation and play an important role in children’s participation in learning activities and their adjustment to school contexts (Acar et al., 2022; Blair and Raver, 2015; McDermott et al., 2002; McDermott et al., 2012). Although approaches to learning have been conceptualized under different labels, attention, effort, self-regulation, and task orientation are widely recognized as core components (Beisly, 2023; Fantuzzo et al., 2007; McWayne et al., 2004).

Learning behaviors are influenced not only by individual characteristics but also by classroom and teacher-related factors; they have also been shown to mitigate the negative impact of adverse classroom environments on academic achievement (Meng, 2015; Shafer and Wanless, 2023). The quality of teacher-child relationships (Hamre and Pianta, 2007) and the emotional support provided by teachers have been found to be associated with children’s approaches to learning (Hu et al., 2017). In addition, learning behaviors can follow different developmental trajectories among children, and these trajectories may be associated with academic difficulties and social–emotional problems (McDermott et al., 2018). These behaviors have also been shown to mediate associations between classroom quality, executive functioning, and children’s academic outcomes (Bustamante and Hindman, 2019; Sung and Wickrama, 2018).

A substantial body of longitudinal and cross-sectional research demonstrates that learning behaviors are closely associated with academic achievement across developmental stages. In particular, these behaviors have been linked to later achievement in reading and mathematics (McClelland et al., 2006; Baker et al., 2015; Beisly et al., 2022; Fitzpatrick and Pagani, 2013; Gullo and Impellizeri, 2022; Li-Grining et al., 2010; McDermott et al., 2011; Razza et al., 2015; Yen et al., 2004; Kitamura et al., 2025; Mercader et al., 2018). Similarly, differences in children’s approaches to learning have been associated with foundational skills, early literacy, and mathematics competencies, with higher levels linked to stronger school readiness (Zhang et al., 2021). Learning behaviors have also been related to broader developmental outcomes, including social competence, epistemic curiosity, and executive functioning (Özcan, 2024; Veziroglu-Celik and Acar, 2020; Guedes et al., 2025). Learning behaviors also provide important cues for teachers when evaluating children’s classroom performance. Behaviors such as attention, persistence, and task orientation are often observable during classroom activities and may inform teachers’ judgments regarding children’s task success or academic competence. For this reason, learning behaviors can be considered not only as individual characteristics that support children’s academic development but also as behavioral indicators that may shape teachers’ interpretations and evaluations of children’s performance.

### The present study

The aim of the present study was to examine how preschool teachers evaluate children’s potential task competence, how these evaluations relate to children’s learning behaviors, and what causal reasoning underlies these judgments, drawing on Weiner’s (1985, 2010) attribution framework. A focus on preschool teachers’ attributions concerning learning and task success was motivated by the relative neglect of this domain in comparison to research on attributions regarding children’s social behavior (Arbeau and Coplan, 2007; Coplan et al., 2011; Coplan et al., 2015; Thijs and Koomen, 2009; Natale et al., 2009). By examining attributional reasoning in a prospective rather than retrospective context, the present study extends prior attribution research beyond post-hoc explanations of observed outcomes to capture the anticipatory causal schemas that teachers bring to children before any task performance occurs, a distinction with direct implications for how learning opportunities are structured in early childhood classrooms. The present study additionally contributes to the attribution literature across cultural contexts, as comparable research in Turkey remains absent. Specifically, the following research questions were addressed:

1. What is the distribution of teachers’ affirmative and negative judgments regarding children’s ability to complete simple and complex tangram tasks?
2. How are teachers’ judgments regarding children’s success in simple and complex tangram tasks distributed jointly?
3. To what extent do children’s overall learning behavior and its subdimensions predict teachers’ binary judgments of task competence when task type is controlled?
4. What types of attributions do teachers make regarding children’s tangram task performance, and do these attributional accounts differ between children in the bottom and top 27% of learning behavior scores?

To address these questions, a convergent parallel mixed-methods design was employed. Teachers evaluated children in their own classrooms on two tangram tasks differing in spatial complexity, tasks those children had not yet attempted, capturing the anticipatory attributional reasoning that may shape learning opportunity structures before performance is observed. This prospective judgment structure distinguishes the present study from prior attribution research, which has predominantly examined teachers’ causal explanations for outcomes already observed (Wang and Hall, 2018). Understanding the basis of teachers’ judgments is important, as these responses may shape children’s understanding of success and failure and their self-evaluations (Koivuhovi et al., 2025).

## Methods

This study employed a convergent parallel mixed-methods design, in which quantitative and qualitative data were collected concurrently, analyzed separately, and integrated during interpretation (Creswell and Plano Clark, 2018). Teachers’ binary judgments regarding children’s task competence were analyzed using generalized estimating equations, while their explanatory statements were examined through directed qualitative content analysis (Hsieh and Shannon, 2005). Integration occurred at the interpretation stage by comparing findings from both strands.

### Participants and procedure

In this study, the sample consisted of 195 preschool children evaluated by their teachers. A total of 57 preschool teachers participated in the study. The study was conducted in Turkey. There were no missing data for any of the demographic variables analyzed. Prior to data collection, ethical approval was obtained from the University Ethics Committee. In addition, written informed consent was obtained from all participating teachers, and participation was voluntary. All participating teachers held a four-year undergraduate degree in Early Childhood Education, ensuring a homogeneous professional training background. Regarding classroom characteristics, 49.7% of the children (*n* = 97) were enrolled in classrooms with 18 or fewer children, whereas 50.3% (*n* = 98) were in classrooms with 19 or more children. In terms of teacher professional experience, 31.8% of the children (*n* = 62) were taught by teachers with 10 years or less of experience, while 68.2% (*n* = 133) were in classrooms led by teachers with 11 years or more experience. The age distribution of the children was as follows: 14.4% (*n* = 28) were 36–48 months, 30.3% (*n* = 59) were 49–60 months, and 55.4% (*n* = 108) were 61 months or older. With respect to gender, 51.8% of the children (*n* = 101) were girls and 48.2% (*n* = 94) were boys, reflecting a balanced gender distribution. Teachers evaluated between 1 and 13 children. All evaluations were provided by the children’s regular classroom teachers, who had worked with the children for at least 3 months prior to data collection. To encourage careful completion of the rating scale and detailed responses to the open-ended items, the number of children rated was not fixed in advance. Teachers were instructed to randomly select at least one child without formally identified developmental conditions from the classroom attendance list and to maintain a balanced representation of girls and boys when selecting additional children. Although teachers received these sampling instructions, adherence to the procedure was not independently verified. This approach was intended to reduce potential selection bias.

### Data analysis

#### Quantitative analysis

Descriptive statistics (mean, standard deviation, minimum, and maximum values) were computed to determine the levels of learning behaviors. Skewness and kurtosis coefficients were examined to assess the normality of the distributions. Descriptive statistics showed total learning behavior scores (skewness = −0.68; kurtosis = −0.65). Subdimensions also fell within the commonly accepted normality limits (skewness and kurtosis values within ±2; George and Mallery, 2010), (skewness = −0.54 to −0.73; kurtosis = −1.01 to 0.46). These distributional properties informed the choice of analytical strategy: because the outcome variable consisted of binary teacher judgments (0 = no, 1 = yes) rather than continuous scores, normality of learning behavior subdimensions was relevant only for characterizing the predictor distributions, not as an assumption of the primary analysis. Descriptive statistics indicated that total learning behavior scores were (M = 66.21, SD = 11.17, range = 37–81). Subdimension means were 25.98 (SD = 5.83) for motivation, 18.74 (SD = 4.78) for attention, and 21.49 (SD = 2.11) for learning attitude (N = 195). Teachers’ binary competence judgments (0 = no, 1 = yes) were analyzed using Generalized Estimating Equations (GEE) with a binomial distribution and logit link function (Liang and Zeger, 1986). Because each child received two evaluations under different task conditions, observations were treated as repeated measures nested within children (*N* = 195; 390 observations). An exchangeable working correlation structure was specified because the two observations per child were expected to be equally correlated regardless of order, reflecting the design in which both task conditions were administered within the same evaluation context. This specification was evaluated against alternative structures (independence, unstructured) using the quasi-likelihood under independence model criterion (QIC) (Pan, 2001), and the exchangeable structure yielded the best fit. Robust (sandwich) standard errors were estimated to obtain population-averaged effects. Model fit was evaluated using QIC, and results are reported as odds ratios (OR) with 95% confidence intervals. To assess multicollinearity among the three learning behavior subdimensions entered simultaneously in Model 2, variance inflation factors (VIF) were examined prior to model interpretation. VIF values ranged from 1.16 to 3.90, indicating no evidence of problematic multicollinearity. Effect sizes were interpreted using odds ratios and corresponding 95% confidence intervals.

### Measures

#### Children’s learning behaviors

Children’s learning behaviors were assessed using the Preschool Learning Behaviors Scale (PLBS; McDermott et al., 2000, 2002). Based on their observations of children’s learning-related behaviors over the previous 2 months, teachers rated the 29 items on a 3-point scale (0 = Not valid, 1 = Sometimes valid, 2 = Most often valid). The Turkish adaptation of the scale was conducted by Veziroğlu Çelik and Acar (2018).

The PLBS comprises three subscales: Attention/Persistence, Competence/Motivation, and Attitude Toward Learning. For the Turkish version, internal consistency coefficients were reported as *α* = 0.88 for Attention/Persistence, *α* = 0.89 for Competence/Motivation, and *α* = 0.71 for Attitude Toward Learning; the overall scale demonstrated an internal consistency of *α* = 0.94 (Veziroğlu Çelik and Acar, 2018; Acar et al., 2022). The internal consistency of the Learning Behaviors Scale was high in the present sample (Cronbach’s *α* = 0.90), indicating reliability.

#### Teacher judgments of children’s task competence

**Figure 1:**
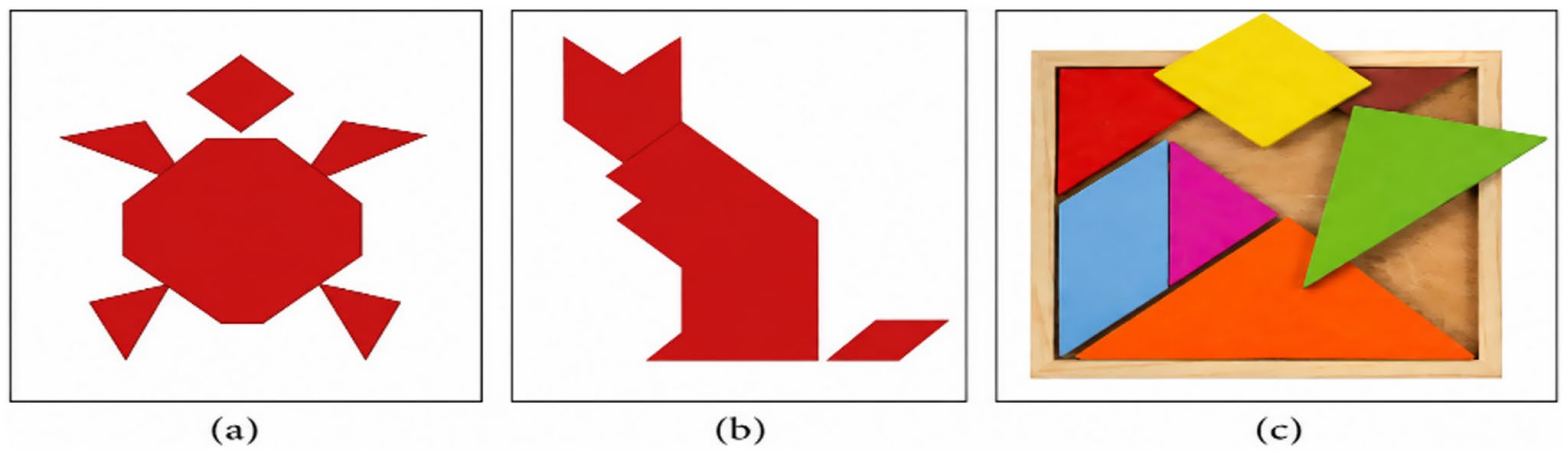
Tangram tasks used in the study. **(a)** Simple task (Turtle figure). **(b)** Complex task (Cat figure). **(c)** The seven standard wooden tangram pieces used in both tasks.

Teachers’ judgments regarding children’s task competence were collected through an online form. Within this form, teachers first completed demographic information and the Preschool Learning Behaviors Scale for each selected child; they subsequently provided their competence judgments and accompanying justifications for the tangram tasks, completing the items for the simple task before those for the complex task. The form was distributed electronically, and all visual materials and instructions were presented digitally within the same interface. For the low-complexity task, teachers were shown a tangram stimulus consisting of (a) the turtle-shaped target model printed on an A4 sheet and (b) the corresponding set of seven standard wooden tangram pieces (see Figure 1). Each tangram piece was proportionally scaled to fit precisely within its designated location on the printed template. The target model and the tangram pieces were displayed together on the same screen. Teachers were provided with the following written instruction within the online form:

*“Do you think this child would be able to complete the turtle-shaped figure drawn on an A4 sheet using the wooden tangram pieces shown below (each tangram piece was proportionally adjusted to fit precisely into its designated place on the A4 template)?* How did you understand this? *What factors influenced your decision regarding whether this child would or would not be able to complete the task?”* Teachers responded to the first question dichotomously (Yes/No), indicating their judgment of the child’s ability to complete the task. These responses constituted the quantitative judgment variable. The second question required an open-ended written response. To identify the basis of teachers’ binary judgments, a follow-up question (“How did you understand this?”) was included. This allowed examination of the reasoning underlying their evaluations rather than focusing solely on outcome-level responses. Teachers completed the form individually. No additional guidance, feedback, or performance cues were provided during the evaluation process.

This study used the tangram task, widely employed in the literature to assess children’s cognitive skills and achievement, to examine teachers’ evaluations of children’s potential task success (Berhenke, 2013; Reiner, 2025). Tangram tasks activate visual–spatial reasoning and part–whole integration processes that are linked to early mathematics achievement (Verdine et al., 2014; Verdine et al., 2017). Evidence also indicates that tangram-based activities are associated with children’s learning engagement and academic performance (Halliday et al., 2018; Weng et al., 2020). Therefore, tangram tasks provide an empirically grounded context for examining teachers’ judgments of children’s cognitive and academic competence. In this study, the two tangram tasks were categorized as low and high complexity based on their structural and spatial properties, as well as prior research using the same materials in a mother–child play context (Işıkoğlu et al., 2025). The materials were used without modification. The turtle figure was identified as a low-complexity task due to its symmetrical and compact structure, limited orientation shifts, and straightforward part–whole integration. Previous findings with children aged 48–72 months showed that most children could complete this figure independently or with minimal maternal assistance, and the task elicited low levels of verbal scaffolding. By contrast, the cat figure was classified as high in complexity because of its asymmetrical and elongated form, the need for more precise angular alignment, and multiple orientation changes during assembly. Earlier results indicated that children had greater difficulty completing this figure independently and required more extensive maternal support. Overall, both structural characteristics and empirical evidence support defining the turtle as lower in cognitive and spatial demand, and the cat as higher in visual–spatial reasoning, working memory, and executive control demands for children in this age range.

## Results

### Quantitative results

Table 1 presents descriptive statistics for learning behavior variables. Total learning behavior scores were (M = 66.21, SD = 11.17). All subdimensions demonstrated acceptable normality (skewness = −0.54 to −0.73; kurtosis = −1.01 to 0.46). Because the primary outcome variable consisted of binary teacher judgments, the distributional properties of learning behavior scores were relevant for characterizing predictor distributions rather than as assumptions of the GEE model, which was specified with a binomial distribution and logit link function appropriate for binary outcomes.

**Table 1:** Descriptive statistics for learning behavior variables (*N* = 195).

Variable	M	SD	Min	Max	Skewness	Kurtosis
Motivation	25.98	5.83	12	33	−0.73	−0.58
Attention	18.74	4.78	8	24	−0.54	−1.01
Learning attitude	21.49	2.11	14	25	−0.72	0.46
Learning behavior (Total)	66.21	11.17	37	81	−0.68	−0.65

#### Distribution and joint structure of competence judgments

Table 2 presents the distribution of teachers’ judgments across task types. A substantially higher proportion of children were evaluated as capable of completing the simple tangram task (74.9%) compared to the complex tangram task (59.0%). Conversely, the proportion of children judged as unable to complete the task increased from 25.1% in the simple condition to 41.0% in the complex condition. These findings indicate that teachers’ competence judgments varied as a function of task complexity.

**Table 2:** Teachers’ judgments of children’s capability to complete simple and complex tangram tasks.

Task type	Yes, *n* (%)	No, *n* (%)
Simple Tangram	146 (74.9%)	49 (25.1%)
Complex Tangram	115 (59.0%)	80 (41.0%)

Table 3 presents the cross-tabulation of teachers’ judgments. An exact McNemar test was used to assess whether teachers’ pairwise judgments differed between simple and complex tangram tasks. The results showed a statistically significant shift in evaluations as task difficulty increased (*p* < 0.001). Examination of discordant pairs revealed that 31 children who were evaluated as competent in the simple task were evaluated as incompetent in the complex task, and no child showed an inverse course, suggesting a strictly unidirectional decrease in perceived competence under higher task demands.

**Table 3:** Cross-tabulation of teachers’ judgments for simple and complex tangram tasks.

Task judgament	Complex: No	Complex: Yes	Total
Simple: No	49	0	49
Simple: Yes	31	115	146
Total	80	115	195

McNemar exact test (binomial distribution), *p* < 0.001.

#### Learning behaviors as predictors of competence judgments

Teachers’ binary competence judgments were analyzed using GEE with a binomial distribution and logit link function, with observations treated as repeated measures nested within children (*N* = 195; 390 observations). An exchangeable working correlation structure was specified because both observations per child were expected to be equally correlated regardless of order, and this specification was supported by QIC model comparison. Robust sandwich standard errors were used to obtain population-averaged effects. Prior to interpretation of Model 2, variance inflation factors confirmed the absence of problematic multicollinearity (VIF range: 1.16–3.90). Table 4 presents the results of two comparative GEE models.

**Table 4:** Comparative GEE models predicting teachers’ positive competence judgments.

Predictor	Model 1: Learning Behavior	OR	p	Model 2: Subdimensions	OR	p
	B			B		
TASK_BIN (0 vs 1)	1.253	3.50	<0.001	1.313	3.72	<0.001
Total learning behavior	0.167	1.18	<0.001	—	—	—
Motivation	—	—	—	0.143	1.15	0.005
Attention	—	—	—	0.253	1.29	<0.001
Learning attitude	—	—	—	−0.060	0.94	0.480
QIC	330.28			322.13		

*N* = 195 children; 390 observations. Exchangeable working correlation structure; robust standard errors. OR, odds ratio.

In Model 1, total learning behavior significantly predicted teachers’ positive competence judgments (B = 0.167, OR = 1.18, *p* < 0.001). Each one-unit increase in total learning behavior was associated with an 18% increase in the odds of being evaluated as competent. Task type was also a significant predictor (B = 1.253, OR = 3.50, *p* < 0.001), indicating that children were substantially more likely to receive a positive judgment for the simple task than for the complex task. Model fit, as indicated by QIC (330.28), provided the reference against which Model 2 was compared.

Model 2 examined the three subdimensions simultaneously. The disaggregated model demonstrated improved fit (QIC reduced from 330.28 to 322.13), indicating greater explanatory precision when behavioral dimensions were considered separately. *Attentional persistence* emerged as the most consistent unique predictor across both significance level and effect size (B = 0.253, OR = 1.29, *p* < 0.001), followed by motivation (B = 0.143, OR = 1.15, *p* = 0.005). Learning attitude did not significantly predict competence judgments (*p* = 0.480). These findings indicate that the aggregate association between learning behaviors and teachers’ judgments is primarily carried by attentional persistence and, to a lesser extent, by motivational behaviors.

### Qualitative results

#### Teachers’ judgments of task performance: an attributional perspective

**Figure 2:**
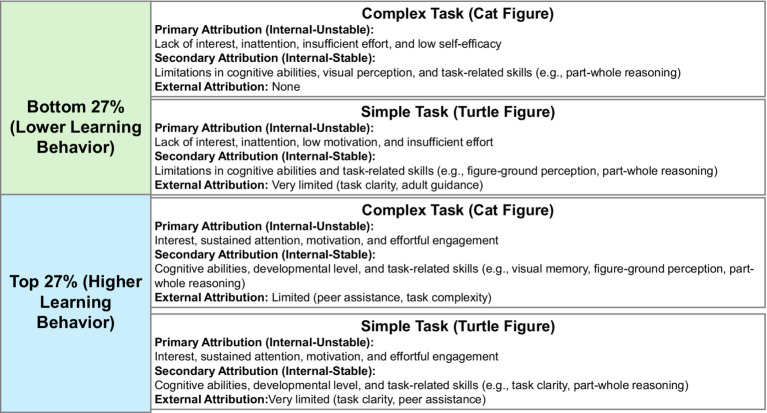
Attributional accounts across learning behavior groups and task complexity conditions.

Teachers’ explanations of children’s task competence were examined within Weiner’s attribution framework across locus (internal–external), stability (stable–unstable), and controllability (controllable–uncontrollable) dimensions. Qualitative comparisons focused on children at the lower (bottom 27%) and upper (top 27%) ends of the learning behavior distribution. This grouping follows Kelley’s (1939) methodological convention for extreme group comparisons in differential research, which maximizes contrast between groups and facilitates identification of theoretically meaningful differences in attributional reasoning. This approach has been adopted in prior teacher perception research examining attributional accounts for children with contrasting behavioral profiles (Arbeau and Coplan, 2007; Coplan et al., 2011). Children in the middle range of the distribution were retained in all quantitative analyses in which the full sample was used. Key content codes included cognitive ability, attention, effort, motivation, self-efficacy, developmental readiness, task-related skills, and support. To facilitate a clearer understanding of these attributional explanations, the findings are first presented visually (see Figure 2) and then described in detail.

### Lower learning behavior group (bottom 27%)

#### Lower learning behavior group: complex task

Teachers judged the majority of children with lower learning behavior scores as incapable of completing the complex tangram task, and they primarily justified these judgments through internal and unstable attributions. The most prominent internal unstable attributions in teachers’ explanations were lack of interest, limited attention, insufficient effort, and low self-efficacy. In addition, it was found that failure in the simple tangram task among children in the lower learning behavior group was particularly justified with reference to interest, attention, and perceptions of self-efficacy. For example: Cannot complete it; attention span is very short and there is a problem with concentration (P36) Cannot complete it because attention is easily distracted in a short time (P10) Cannot complete it. Interest in this type of material is very limited (P19). Moreover, some teachers indicated that lack of attention and interest, together with low self-efficacy, functioned as barriers preventing the child’s existing capacity from being translated into successful performance. For example: Can complete it, but attention span is very short, especially when the child thinks he/she has made a mistake, and gives up quickly (P9) A very active and intelligent child who learns many concepts faster than peers, but has a short attention span (P12) Could do it with support if he/she tried, but would not want to do it because of general unwillingness toward everything (P1) Would make an effort, but would frequently seek adult opinion and approval. When no answer was received, the child would stop trying and move on to another toy (P6).

The second prominent attribution reflected in teachers’ explanations was limitation in cognitive ability, intelligence, development, and capacity to possess the knowledge and skills required by the task, such as visual perception and part-whole relations. For example: Not cognitively mature enough (P13) Limited visual and logical ability (P47) Cognitive development is far below the level expected for age (P49). Only a few teachers judged these children as capable of completing the task, and these judgments were justified through internal and stable attributions, such as cognitive ability, intelligence, developmental level, and the capacity to possess the knowledge and skills required by the task, including part-whole relations. For example: Has the competence to adapt part-whole relations to different situations (P37) Intelligence and perception are good (P23). Teachers in this group did not refer to external attributions.

#### Lower learning behavior group: simple task

Teachers judged a substantial proportion of children with lower learning behavior scores as incapable of completing the simple tangram task, and they primarily justified these judgments through internal and unstable attributions. The most salient internal unstable attributions in teachers’ explanations were lack of interest, limited attention, low motivation, and insufficient effort. For example: Cannot complete it. Makes an effort, but cannot reach the result. Attention becomes distracted (P38) At a level where he/she could do it, but unless willing, attention is constantly distracted and the child postpones doing it (P1) Cannot complete it; does not like toys with pieces (P9) Has difficulty doing it. A child who gives up easily. In activities, usually says “I cannot do this” and stops trying (P48). The second prominent attribution reflected in teachers’ explanations was internal and stable attributions referring to limitations in cognitive ability, intelligence, development, and the capacity to possess the knowledge and skills required by the task, such as visual perception and part-whole relations. For example: Not cognitively mature enough (P13) Limited visual and logical ability (P47) Because the child is far below the level expected for age (P49).

For some children in this group, teachers judged these children as capable of completing the task, and these judgments were justified through internal and stable attributions, such as cognitive ability, intelligence, developmental level, and the capacity to possess the knowledge and skills required by the task, including part-whole relations. For example: Has the competence to adapt part-whole relations to different situations (P37) Intelligence and perception are good (P23). In addition, teachers in this group also referred, although to a limited extent, to external attributions. For example: May be able to complete this figure because the shapes are more clearly defined, so the child may decide more easily which piece to place (P2) Could do it with help. I think the child could complete it if guided by an adult (P19) Could succeed because the shapes are clearer (P42).

Overall, teachers generally judged children in the lower learning behavior group as incapable of completing the tangram task. In this group, negative competence judgments were justified through internal attributions. Among these explanations, internal unstable attributions were emphasized more than internal stable attributions. On the other hand, it is noteworthy that when justifying negative competence judgments for children in this group, they made no reference to external attributions (e.g., task, teacher, family, environment) in the difficult task and only very limited reference to such attributions in the easy task.

### Higher learning behavior group (top 27%)

#### Higher learning behavior group: complex task

Teachers judged the majority of children with higher learning behavior scores as capable of completing the complex tangram task, and they justified these judgments through internal and unstable attributions. The most salient internal unstable attributions in teachers’ explanations were having interest, attention, motivation, and making effort. Teachers in this group often combined internal stable and internal unstable characteristics when justifying why a child would succeed in the tangram task. For example: Can complete it. If there is difficulty, the child can also ask for help. General development is above the class level (P1) Can complete it; intellectual development is at the expected level and the child does not give up easily (P11). Unlike the lower learning behavior group, in the higher learning behavior group teachers placed particularly strong emphasis on effort when justifying success in completing the tangram figure, more so than other internal unstable tendencies. For example: Makes an effort to do it and, if necessary, asks for help. Even if help is needed, I am sure the child will still make an effort (P8) Can complete it; if unsuccessful in the first attempts, the child tries again and again and eventually completes the tangram. My decision was influenced by the child’s repeated attempts during free play in the classroom, especially while building with blocks. When the blocks collapsed, the child repeatedly tried again until constructing the desired structure (P56) Can complete it because interest in tangram activities during free play in the classroom is quite high, and the child is very willing to construct things (P7) Can complete it; very attentive and detail-oriented (P39) Can complete it. The child generally sustains attention during activities, is patient, and is willing to fulfill given tasks (P27). The second prominent attribution reflected in teachers’ explanations was internal stable attributions. For some children in this group, teachers’ positive competence judgments were justified through internal stable attributions, such as cognitive ability, intelligence, positive development, and the capacity to possess the knowledge and skills required by the task, including part-whole relations. For example: Yes, can complete it. I think the child has the sufficient cognitive level to complete this activity (P13) Can complete it. Visual memory is good and hand-eye coordination is very good (P39) I know the child has visual intelligence. Therefore, the child will complete this task without difficulty (P43).

Negative competence judgments were infrequent in this group and were justified through internal stable attributions. For example: Cannot complete it because cognitively inadequate (P12) Because cognitive development and visual memory are not at an advanced level (P49). In justifying their negative judgments, teachers also made limited reference to external stable and unstable attributions. For example: Cannot complete it fully without help. Can produce something similar. In such activities, the child asks peers for help (P38) May have difficulty. If it were made up of different colors, the child could do it (P44) It is a complex shape, but the child could complete it with some support (P2).

#### Higher learning behavior group: simple task

Teachers judged the vast majority of children with higher learning behavior scores as capable of completing the simple tangram task, and they justified these judgments through internal and unstable attributions. The most salient internal unstable attributions in teachers’ explanations were having interest, attention, motivation, and making effort. For example: Can complete it. Attention span is slightly longer than that of peers (P27) Yes, can do it. Does not like giving up and is open to new activities (P46) Can complete it because interest in tangram activities is quite high (P7) Can do it without help. Developmental level is good. Effort, willingness, and level of intelligence are all suitable for accomplishing it (P1). The second prominent attribution reflected in teachers’ explanations was internal stable attributions. For some children in this group, teachers’ positive competence judgments were justified through internal stable attributions, such as cognitive ability, intelligence, positive development, and the capacity to possess the knowledge and skills required by the task, including part-whole relations. For example: Can complete it; very good at understanding figure-ground relations (P8) Has thinking skills (P24) Can do it. Good understanding of shape and direction relations and knows that, for example, two triangles make a square, and can also place a triangle in different ways (P33) Visual memory and attention span are high (P36) Cognitive development and visual memory are at an advanced level (P49). In justifying their positive judgments, teachers also made limited reference to external stable and unstable attributions. For example: Can complete it. Here the figure is clearer, and the child can do it simply by making a few changes to the pieces in hand (P33). Negative competence judgments were infrequent in this group and were justified through external unstable attributions. For example: Cannot fully complete it without help. Can produce something similar. In such activities, the child asks peers for help (P38).

## Discussion

In this study, we found that preschool teachers’ competence judgments regarding children’s ability to complete the tangram tasks were shaped by both task complexity and teachers’ perceptions of children’s learning behaviors. As task complexity increased, the proportion of children judged as competent decreased, and this shift was strictly unidirectional. Among the teacher-rated learning behavior dimensions examined, attentional persistence emerged as the strongest predictor of positive competence judgments, whereas learning attitude did not make a significant independent contribution. We also found that teachers explained their judgments regarding whether a child could complete the tangram task predominantly through internal child-related attributions. Among children with lower teacher-rated learning behavior scores, anticipated failure was commonly attributed to inattention, low effort, developmental immaturity, and limited cognitive ability, whereas among children with higher teacher-rated learning behavior scores, anticipated success was primarily explained through effort, persistence, and ability. By contrast, contextual and instructional factors were rarely referenced, regardless of task condition or learning behavior group. The findings extend attribution research by showing that attributional explanations are evident not only after performance has occurred but also when teachers evaluate children’s likely success before they have attempted a task. Taken together, the results suggest that preschool teachers’ competence judgments regarding the tangram tasks are closely linked to teachers’ perceptions of children’s learning behaviors and are accompanied by attributional explanations that emphasize child-level characteristics over contextual factors.

### Task complexity and the distribution of competence judgments

The first and second research questions addressed how teachers’ positive and negative judgments were distributed across simple and complex task conditions and how these judgments co-varied at the level of individual children. The quantitative findings of this study showed that as task complexity increased from the simple to the complex tangram, a substantially higher proportion of children shifted from a positive to a negative competence evaluation, and this change was statistically significant. Notably, no child followed the reverse course, indicating a strictly unidirectional shift in judgment as task demands increased. This finding may have implications beyond the immediate evaluation context. Although not directly examined in the present study, prior research theoretically suggests that teachers’ competence judgments may extend beyond specific task assessments and shape broader expectations about children’s abilities, which in turn may influence how children come to understand their own success and failure (Koivuhovi et al., 2025). This result aligns with the broader teacher judgment literature showing that harder tasks produce more conservative and sometimes less accurate predictions (Südkamp et al., 2012). The complex tangram introduces visual–spatial and working-memory demands that may exceed what teachers can reliably infer from naturalistic classroom observation (Verdine et al., 2014, 2017), potentially increasing reliance on general behavioral impressions rather than task-specific evidence. An alternative interpretation concerns the cognitive experience of the teacher as evaluator. When a stimulus is visually complex, teachers may perceive the task as more demanding and this perception may contribute to more conservative competence judgments.

### Learning behaviors as predictors of competence judgments

The third research question asked how children’s learning behaviors predicted teachers’ competence judgments after controlling for task type. The quantitative findings of this study established that total learning behavior score was a significant predictor of positive competence evaluation. When the three subscales were examined simultaneously, model fit improved, and attentional persistence emerged as the most consistent predictor, followed by motivation. Learning attitude did not reach significance. Because both the learning behavior ratings and the competence judgments were provided by the same teachers, these associations are interpreted as relationships between teachers’ perceptions of children’s learning behaviors and teachers’ own competence judgments, rather than as direct associations between children’s actual behavior and their competence. The prominence of attentional persistence is theoretically coherent. Attention is among the most directly observable dimensions of children’s classroom behavior and has been identified as a proximal contributor to early academic outcomes (Duncan et al., 2007; McClelland et al., 2006). In line with teacher belief research, teachers appear to rely on observable behavioral indicators, particularly attention and perseverance, when forming judgments about children’s task competence (Pajares, 1992; Fives et al., 2015). This emphasis on attention is consistent with research highlighting the role of behavioral engagement in children’s learning processes (Fredricks et al., 2004) and with evidence that teachers frequently interpret children’s competence and success in terms of attentional processes (Mao and Grammer, 2026). Teachers who regularly observe a child sustaining focus during structured activities accumulate behavioral evidence that informs their sense of that child’s competence. The reliance on attentional persistence as a judgment cue may therefore reflect a reasonable inference about task persistence. Longitudinal evidence supports this inference: Tõeväli and Kikas (2016) found that children’s task persistence predicted teachers’ subsequent ability attributions for success and also predicted children’s later math performance independently, suggesting that teachers’ attention to persistence as a judgment cue tracks a behavioral characteristic that is genuinely consequential for children’s academic outcomes. Furthermore, teachers’ attributions for children’s achievement have been shown to be associated with children’s subsequent achievement outcomes (Upadyaya et al., 2012; Upadyaya and Eccles, 2015), and such attributions are influenced by children’s motivation and performance while also relating to their later motivational and performance trajectories (Natale et al., 2009). Consistent with this, Natale et al. (2009) showed that children’s reading motivation and performance predicted teachers’ subsequent ability and effort attributions, suggesting that the present findings extend this child-to-teacher effect to a spatial-constructive task domain and a prospective judgment context. These findings suggest that the weight teachers assign to attention as a judgment cue may carry implications that extend beyond immediate competence evaluation. At the same time, this reliance risks conflating observable engagement with domain-specific spatial ability. Children who face attentional challenges in group settings may possess strong visual–spatial reasoning that remains invisible to teachers whose judgments rest heavily on sustained focus, and for these children competence estimates based on behavioral observation alone may consistently fall below their actual capacities. Whether teachers’ reliance on attentional persistence as a judgment cue is best characterized as a reasonable heuristic or a rationally contextualized inference is a question that the present data cannot resolve definitively. The evidence is consistent with both readings: it is reasonable in that attentional persistence genuinely predicts academic outcomes (Tõeväli and Kikas, 2016), and it may be rationally contextualized in that teachers who know a child well may draw on accumulated classroom evidence rather than applying a generic heuristic.

### Attributional reasoning across learning behavior groups and task conditions

The fourth research question asked which causal attributions teachers invoked to explain their judgments about children’s ability to complete the tangram tasks, and whether these attributions differed between children at the lower and upper ends of the learning behavior distribution. Content analysis, guided by Weiner’s (1985, 2010) attribution theory, revealed several theoretically meaningful tendencies in teachers’ attributional reasoning.

The qualitative findings of this study showed that internal attributions were dominant among children with both lower and higher teacher-rated learning behavior scores, as well as across both the simple and complex tangram task conditions. References to external factors, including task structure, instructional scaffolding, and environmental support, were infrequent overall and appeared almost exclusively in teachers’ reasoning about the simpler task. In educational contexts, such reasoning may reinforce interpretations of children’s competence that emphasize child-level characteristics over contextual influences. According to Weiner’s (1985, 2010) framework, stable internal attributions for failure are associated with reduced expectancy of success and diminished investment in instructional support. The present qualitative findings extend this theoretical perspective by documenting that such attributions were produced prospectively, before performance was observed, suggesting that causal schemas formed prior to evaluation may shape teachers’ anticipatory judgments before children have an opportunity to demonstrate their capacities. This prospective dimension is particularly consequential when considered alongside Rosenthal and Jacobson’s (1968) foundational demonstration that teacher expectations can function as self-fulfilling prophecies, influencing actual child outcomes through differential instruction and feedback. When internal attributions for anticipated failure are formed in advance and remain unexamined, prior research suggests they may have implications for expectancy-related processes (Rosenthal and Jacobson, 1968; Jussim and Harber, 2005). Although the present study did not examine instructional behaviors or classroom interactions, these findings are theoretically relevant for understanding how anticipatory judgments might relate to such processes. Within the group of children with lower learning behavior scores, the qualitative findings of this study indicated that failure attributions combined references to stable internal characteristics, such as limited cognitive ability and developmental immaturity, with unstable internal characteristics, including inattention, insufficient effort, and low motivation. This combination suggests that teachers considered not only children’s observable engagement during the task but also their perceived cognitive competence, including visual perception and part-whole reasoning skills, a finding consistent with Fantuzzo et al. (2004). This co-occurrence is theoretically notable. Weiner’s model predicts that unstable attributions for failure should preserve some degree of optimism about future change, because transient and controllable factors are in principle amenable to intervention. However, the qualitative evidence indicates that this optimistic inference did not materialize in teachers’ reasoning about this group. Attentional and motivational limitations were described not as transient states but as behaviorally confirmed tendencies that teachers had observed repeatedly across classroom contexts. In this way, what the attribution model classifies as unstable factors were treated in practice as empirically settled characteristics of the child. The very limited use of external attributions, especially for the difficult task, further suggests that failure was interpreted largely in terms of child-related characteristics, a finding consistent with evidence that early childhood educators tend to explain children’s behaviors primarily through child-internal factors, even in the context of challenging behavior (Panthi et al., 2026). It is also worth noting that children who engage more actively in challenging tasks are more likely to demonstrate persistence and task-focused behavior (Halliday et al., 2018), which may explain in part why low behavioral engagement was interpreted by teachers as a reliable signal of likely task failure. This finding represents an important qualification of the attribution-expectancy link that Weiner’s model predicts and warrants further investigation in research on teacher cognition in early childhood.

A particularly consequential finding concerns the risk of compounded disadvantage for children who are already identified as having lower learning behavior profiles. The quantitative findings of this study demonstrated that children at the lower end of the learning behavior distribution were substantially more likely to receive negative competence evaluations on hypothetical tangram tasks. The qualitative findings of this study further revealed that teachers accounted for these negative evaluations almost exclusively through internal attributions, without reference to instructional strategies, classroom arrangements, or other modifiable contextual factors. When children who are already at a relative disadvantage in terms of observable learning behaviors are simultaneously evaluated as unlikely to succeed on cognitively demanding tasks, and when teachers explain these anticipated failures primarily through fixed or habitual child-level characteristics, these patterns may have implications for instructional opportunities and expectancy-related processes. These findings suggest a theoretically grounded risk of dual disadvantage: children with lower learning behavior scores received fewer positive competence judgments and were simultaneously evaluated predominantly through internal attributions. Drawing on prior research (Brophy and Good, 1970; Gentrup et al., 2020), this configuration may carry implications for instructional opportunities and expectancy-related processes; however, these pathways were not directly tested in the present study. Research on teacher attributions indicates that when low achievement is attributed to low ability, teachers tend to respond with pity, whereas attributing low achievement to low effort may elicit greater frustration or reduced helping behavior (Georgiou et al., 2002). Whether mediated through emotional responses or through reduced instructional investment, such dynamics may shape the quality of teacher-child interactions and limit the learning opportunities available to children already perceived as less capable. Evidence from a person-centered study of teacher attribution profiles further supports this concern: teachers whose explanations of student failure centered on external and uncontrollable factors showed the lowest levels of helping behavior intention, suggesting that reduced instructional investment can emerge through multiple attributional routes (Brun et al., 2022). In the present study, failure attributions for children with lower teacher-rated learning behavior scores were predominantly internal rather than external; however, the limited reference to modifiable contextual factors in these accounts raises a parallel concern, as attributions that position failure as a characteristic of the child may similarly reduce the perceived value of instructional intervention.

Research on teacher expectations suggests that children perceived as less capable tend to receive less cognitively stimulating instruction and fewer opportunities to demonstrate competence (Brophy and Good, 1970; Gentrup et al., 2020; Jussim and Harber, 2005). Although instructional behavior was not examined in the present study, the attributional patterns identified here may be relevant to theoretical accounts of how teacher judgments and expectations relate to children’s educational experiences. The near-complete absence of external attributions in teachers’ failure accounts for this group warrants explicit attention in professional development contexts. From an equity standpoint, children whose learning behavior profiles already place them at a relative disadvantage may face an additional, attribution-mediated barrier to receiving the instructional support they need: not because teachers are indifferent, but because the causal schemas available to them do not naturally generate instructional responses. Teachers’ reasoning about children with higher learning behavior scores showed a different composition. Success attributions for this group integrated internal stable and internal unstable factors in a complementary way, with particular emphasis on effort. The greater emphasis on effort in success attributions is consistent with evidence that higher levels of task motivation are associated with greater attribution to ability and effort (Natale et al., 2009). The more differentiated explanatory accounts in the group with higher teacher-rated learning behavior scores, in which a small number of teachers acknowledged task-level features and the potential role of peer support, contrast with the more person-focused reasoning observed for children with lower teacher-rated learning behavior scores. This contrast suggests that teachers’ explanations were not distributed uniformly across children but varied according to prior behavioral impressions. In particular, contextual factors appeared less frequently in teachers’ explanations concerning children perceived as less behaviorally engaged.

### Integrating quantitative and qualitative findings

The convergent parallel design of this study enables a more complete account of teachers’ competence judgments than either analytic strand could provide alone. The quantitative findings established that attention/persistence was the most consistent predictor of teachers’ judgments across task conditions and that task complexity independently and substantially reduced the likelihood of a positive evaluation. The qualitative findings provide an explanatory account of these associations. The prominence of attention/persistence as a quantitative predictor is directly mirrored in the qualitative data: teachers who judged children as capable or incapable consistently referenced attention, persistence, and engagement as primary reasons for their competence judgments, particularly for children at the lower end of the teacher-rated learning behavior distribution. This alignment between the statistical weight assigned to attention/persistence and its salience in teachers’ spontaneous explanations suggests convergent validity across the two analytic strands rather than a methodological artifact. The effect of task complexity on teachers’ judgments is also illuminated by the qualitative findings. The quantitative evidence showed that a substantially higher proportion of children were evaluated negatively under the complex task condition. The qualitative evidence shows how teachers’ attributional reasoning accompanied this shift: as task demands increased, teachers of children with lower teacher-rated learning behavior scores made virtually no reference to external or contextual factors and instead relied on the same child-internal characteristics they had invoked for the simpler task. Although judgments became more negative under the complex condition, this reasoning remained largely similar across task conditions. Taken together, the quantitative and qualitative findings suggest that task difficulty was associated with less favorable competence judgments, whereas teachers’ explanations continued to emphasize child-level characteristics across task conditions.

### Limited external attributions

A notable finding of this study is the near-total absence of structural and instructional factors in teachers’ explanatory accounts. The qualitative data indicate that teachers make minimal reference to teacher support and task characteristics when justifying their judgments about whether children can or cannot perform the hypothetical task, and no reference to other potential influences on children’s performance, including characteristics of the learning environment, the teaching process, the curriculum, and instructional materials. This finding is consistent with prior evidence demonstrating that, despite structural variables accounting for significant variance in achievement outcomes, there remains a prevailing tendency to attribute children’s academic performance to child-level factors (Georgiou et al., 2002). The near-exclusive reliance on child-level attributions in teachers’ explanations suggests that children’s potential competence may be constructed primarily as an individual characteristic (Chen and McNamee, 2011).

This interpretation warrants closer scrutiny. Learning behaviors are not solely a product of intrinsic characteristics; they are substantially shaped by contextual factors such as teacher support, peer interactions, and the broader classroom environment. Supportive teacher–child interactions and well-organized, high-quality early childhood classrooms have been shown to enhance children’s task-related engagement, sustained attention, and interaction (Hamre et al., 2007; Williford et al., 2013; Bustamante and Hindman, 2019). Emotionally supportive learning environments, in particular, may elicit precisely the learning behaviors that teachers interpret as indicators of competence. The absence of such contextual factors from teachers’ attributional accounts thus points to a gap in attribution repertoire, one that professional development frameworks could be positioned to address. Research on teacher beliefs indicates that reorienting attribution patterns requires sustained, structured professional learning rather than incidental exposure (Fives et al., 2015; Sabarwal et al., 2022). The open-ended design of the present study afforded teachers equal opportunity to reference child-level, teacher-level, or contextual factors; nonetheless, self-directed attributions were virtually absent. This finding may resonate with Lauermann’s (2023) observation that the underrepresentation of teacher-directed explanations in the literature reflects not only how attribution research is designed, but also how teachers themselves conceptualize children’s competence, a conceptualization that, in the present study context, appears to be largely decontextualized from instructional conditions.

## Conclusion

This study examined how preschool teachers evaluate children’s likely competence in tangram tasks before task performance is observed and what attributional explanations accompany these evaluations. The findings showed that competence judgments were sensitive to both task complexity and teachers’ perceptions of children’s learning behaviors. As task complexity increased, fewer children were evaluated as capable, and this shift was unidirectional. Among the learning behavior dimensions, Attention/Persistence emerged as the most consistent predictor of positive competence judgments. Qualitative findings further indicated that teachers predominantly explained their evaluations through internal child-related factors, particularly for children with lower teacher-rated learning behavior scores, while contextual and instructional factors were mentioned only rarely. The study contributes to attribution research by demonstrating that Weiner’s attribution framework can be applied to prospective judgment contexts. Whereas previous studies have largely examined teachers’ explanations of observed outcomes (Wang and Hall, 2018), the present findings indicate that similar attributional patterns may also be evident when teachers evaluate children’s likely success before they have attempted a task. The findings also extend research on teacher judgment in early childhood settings, which has focused primarily on language, literacy, and numeracy domains (Baker et al., 2015; Kilday et al., 2012), to spatial-constructive task contexts. These conclusions should be interpreted in light of several limitations, including the hypothetical task design, the absence of direct performance measures, and the lack of observational data on teachers’ instructional practices. Consequently, the study cannot determine how competence judgments influence subsequent instructional behavior or children’s later performance. Future research should examine how prospective attributional explanations relate to observed instructional practices and children’s actual task performance in classroom settings.

## Limitations and directions for future research

Several features of the present study should be noted when interpreting its findings. First, competence judgments were based on hypothetical task evaluations rather than observed performance. Although this design enabled standardized stimulus presentation and direct comparison across difficulty conditions, teachers had no task-specific performance data on which to anchor their evaluations. The associations between learning behaviors and competence judgments documented in the quantitative findings therefore likely reflect the weight of general behavioral impressions under conditions of evaluative uncertainty and may not fully generalize to contexts in which direct performance evidence is available (Wang and Hall, 2018). Second, both the predictor and outcome variables were derived from the same source, as teachers provided both the learning behavior ratings and the competence judgments. Shared method variance may have inflated the observed associations (Podsakoff et al., 2003). Future research incorporating observer-rated or directly assessed learning behaviors would help provide a more conservative estimate of the relationship between children’s behavioral profiles and teachers’ evaluations. Third, the study did not control for contextual variables known to be associated with both children’s learning behaviors and teachers’ evaluative tendencies, including family socioeconomic status and classroom quality. If children from less advantaged backgrounds tend to display lower learning behavior scores and if teachers in lower-resourced classrooms are more likely to form negative competence judgments, the observed associations may partly reflect socioeconomic confounding rather than a direct relationship between behavioral engagement and teacher evaluation. The absence of these controls also extends to the qualitative strand: classroom-level factors such as group size and teacher experience varied meaningfully across the sample but did not surface in teachers’ spontaneous explanations, a finding that itself warrants interpretation but that limits the conclusions that can be drawn about structural influences on attributional reasoning. Future research incorporating family background variables and classroom quality indices would enable a more precise estimate of the independent contribution of children’s learning behavioral profiles to teachers’ judgments.

Fourth, the qualitative analysis concentrated on children at the extremes of the learning behavior distribution, and the online questionnaire format may have constrained the depth of teachers’ open-ended responses relative to what semi-structured interviews or stimulated recall procedures might elicit (Patton, 2015). The findings for teachers’ attributional reasoning therefore reflect the most contrastive cases in the sample and may not capture the full range of explanatory accounts teachers produce for children with moderate behavioral profiles. Additionally, the focus on tangram tasks, which draw primarily on visual–spatial reasoning, limits generalizability to other cognitive domains; the role of attentional persistence as a judgment cue may be more pronounced for tasks in which sustained focus is particularly visible, and future research examining teacher judgments across multiple task domains would clarify whether the present findings reflect domain-general or domain-specific processes.

Finally, the study did not include a measure of children’s actual task performance, and it did not examine whether teachers’ attributional accounts translate into differential instructional behavior. Without an accuracy criterion, it is not possible to assess whether teachers’ judgments were well calibrated to children’s spatial competence or to test the attribution-to-instruction pathway that the dual disadvantage argument implies. The practical implications of the present findings therefore rest on theoretically grounded rather than empirically verified assumptions about the relationship between causal reasoning and instructional investment. Linking teachers’ prospective judgments and attributional accounts to direct performance assessments and observational measures of instructional behavior in future research would provide a more complete test of the mechanisms proposed here and enable accuracy analyses in the sense outlined by Südkamp et al. (2012).

## Data Availability

The datasets generated and analyzed during the current study are not publicly available due to ethical restrictions but are available from the corresponding author on reasonable request. Requests to access the datasets should be directed to aadak@pau.edu.tr.
